# Adaptive Regulation of Stopover Refueling during Bird Migration: Insights from Whole Blood Transcriptomics

**DOI:** 10.1093/gbe/evad061

**Published:** 2023-04-17

**Authors:** Anastasios Bounas, Chrysoula Komini, Artemis Talioura, Elisavet-Aspasia Toli, Konstantinos Sotiropoulos, Christos Barboutis

**Affiliations:** Department of Biological Applications and Technology, University of Ioannina, Greece; Department of Biological Applications and Technology, University of Ioannina, Greece; Department of Biological Applications and Technology, University of Ioannina, Greece; Department of Biological Applications and Technology, University of Ioannina, Greece; Department of Biological Applications and Technology, University of Ioannina, Greece; Department of Biological Applications and Technology, University of Ioannina, Greece; Antikythira Bird Observatory, Hellenic Ornithological Society/BirdLife Greece, Athens, Greece

**Keywords:** candidate genes, differential gene expression, metabolism, phenotypic plasticity, RNA-seq, water homeostasis

## Abstract

Migration is one of the most energy-demanding tasks in avian life cycle. Many birds might not have sufficient fuel stores to cover long distances, so they must stop to rest and refuel at stopover sites, especially after the crossing of large ecological barriers. There, birds undergo several behavioral, morphological, and physiological trait adjustments to recover from and prepare for their journey; however, regulation of such processes at the molecular level remains largely unknown. In this study, we used transcriptomic information from the whole blood of migrating garden warblers (*Sylvia borin*) to identify key regulatory pathways related to adaptations for migration. Birds were temporarily caged during spring migration stopover and then sampled twice at different refueling states (lean vs. fat), reflecting different migratory stages (stopover arrival vs. departure) after the crossing of an extended ecological barrier. Our results show that top expressed genes during migration are involved in important pathways regarding adaptations to migration at high altitudes such as increase of aerobic capacity and angiogenesis. Gene expression profiles largely reflected the two experimental conditions with several enzymes involved in different aspects of metabolic activity being differentially expressed between states providing several candidate genes for future functional studies. Additionally, we identified several hub genes, upregulated in lean birds that could be involved in the extraordinary phenotypic flexibility in organ mass displayed by avian migrants. Finally, our approach provides novel evidence that regulation of water homeostasis may represent a significant adaptive mechanism, allowing birds to conserve water during long-distance flight, mainly through protein catabolism.

**Significance**

Despite the wealth of information available about migration physiology, there is little known about how migration is seasonally controlled at the molecular level. Comparing gene expression at specific stages of avian migration can allow the identification candidate genes and potential regulatory pathways. However, sampling for tissue-specific gene expression is often destructive, so the same individuals cannot be assessed at different states. Here, using a whole blood transcriptomic approach, we were able to identify genes that were previously found to be expressed in specific tissues and furthermore provide additional genes involved in a wide range of functions and identify novel pathways that represent significant adaptive mechanisms such as migration at high altitudes, phenotypic flexibility in organ mass, and water conservation during long-distance flight.

## Introduction

Every year, billions of birds cover enormous distances between their breeding and wintering grounds. Migration is an integral part of the annual cycle of many bird species, evolved as an adaptive response to seasonal environments, allowing birds to take advantage of spatially segregated, seasonally abundant food resources (Newton 2007; Pulido 2007). Those seasonal movements regularly involve the crossing of vast ecological barriers such as deserts, mountain ranges, and large water bodies. In the western Palaearctic migration system, most trans-Saharan passerine migrants have to cross both the Sahara Desert and the Mediterranean Sea that can be considered as one combined ecological barrier because there are hardly any possibilities to refuel during crossing (Biebach 1990). Therefore, birds should be energetically prepared for this demanding task (Fransson et al. 2008; Bayly et al. 2012).

The increased energetic demands associated with migration can be a major obstacle for many birds as they might not have sufficient fuel stores to cover long distances, so they must stop and replenish their energy reserves periodically. Stopovers are an important component of migration allowing birds to rest and refuel. Nevertheless, they must be precisely timed and coordinated to maximize fitness; birds must efficiently deposit fuel and at the same time maintain an optimal travel schedule that allows them to arrive early at the breeding grounds (Alerstam and Lindström 1990; Alerstam 2011). However, the decision of when to stop is not always facultative. Large barriers such as the Mediterranean Sea and the Sahara Desert can force a migrant to a nonstop flight until reaching the other side. Passerines arriving with severely depleted fuel reserves, after crossing this large ecological barrier, have been reported in central and eastern areas of the Mediterranean (Moreau 1969; Barboutis et al. 2022). Undoubtedly, the most obvious and perhaps the most important function of stopovers is to accumulate energy, allowing depleted birds to resume their flight after building fat and protein fuel reserves that have been used (Jenni and Jenni-Eiermann 1998; Schwilch et al. 2002). However, stopovers can also serve as periods during which migrants recover physiologically from the preceding migratory endurance flight including muscle repair, organ recovery, hyperthermia, water stress, oxidative balance, and constitutive immune function (Guglielmo et al. 2001; Eikenaar, Hegemann, et al. 2020a; Schmaljohann et al. 2022).

To prepare for migration, a significant number of behavioral, morphological, and physiological trait adjustments must be made to develop the so-called migration phenotype (Åkesson and Hedenström 2007; Ramenofsky and Wingfield 2007). These changes are characterized by a high degree of phenotypic flexibility, which is the norm rather than the exception in migratory birds (Piersma and Drent 2003). Before departure, birds develop hypertrophy of heart, flight and skeletal muscles, and organs that can support endurance flight (Piersma 1998) and are reduced in size by catabolism during the journey, thus providing fuel through amino-acid deamination (Jenni and Jenni-Eiermann 1998; Bauchinger and Biebach 2005). The main sources of energy required to support bird flight include lipids, proteins, ketone bodies, and carbohydrates. Multiple biochemical alterations are involved to the transport and metabolism of these fuels (Landys et al. 2005), whereas organs that augment feeding including the gizzard, intestine, liver, and kidneys enlarge during stopovers but shrink during flight when food intake and digestion are inactive (Landys-Ciannelli et al. 2003). In cases that individuals migrate long distances without available water sources, osmoregulatory adjustments are further required to conserve water. Lipid oxidation and protein catabolism are the two main processes that may contribute to water balance (Ramenofsky and Wingfield 2007).

Despite the wealth of information available about migration physiology, there is little known about how migration is seasonally controlled at the molecular level. The advance of RNA sequencing technology made it possible to actually investigate the differential expression of genes across the genome of migratory and resident individuals or seasonal differences of gene expression in migratory birds, targeting a better understanding of the genetic underpinnings of bird migration (Kraus and Wink 2015; Merlin and Liedvogel 2019). Comparing gene expression at specific stages of avian migration provides an opportunity to identify a large number of genes and potential regulatory pathways involved in important aspects of a species’ life history.

In this study, we used transcriptomic information from whole blood of migrating garden warblers (*Sylvia borin*) to identify key regulatory pathways related to adaptations for migration. Birds were temporarily caged during spring migration under ad libitum food conditions and then sampled twice at different refueling states (lean vs. fat), reflecting different migratory stages (stopover arrival vs. departure) after the crossing of the extended ecological barrier formed by the Sahara Desert and the Mediterranean Sea, in order to identify genes potentially associated with fuel accumulation and/or recovery of physiological processes. This approach will allow us to gain new insights into the molecular mechanisms controlling migration. Except from identifying candidate genes for future functional studies and orthologous gene groups that could be of interest in human metabolic studies, our results could be used to significantly aid the conservation of this fascinating albeit disappearing (Wilcove and Wikelski 2008) aspect of avian life, migration.

## Results

**Fig. 1.**
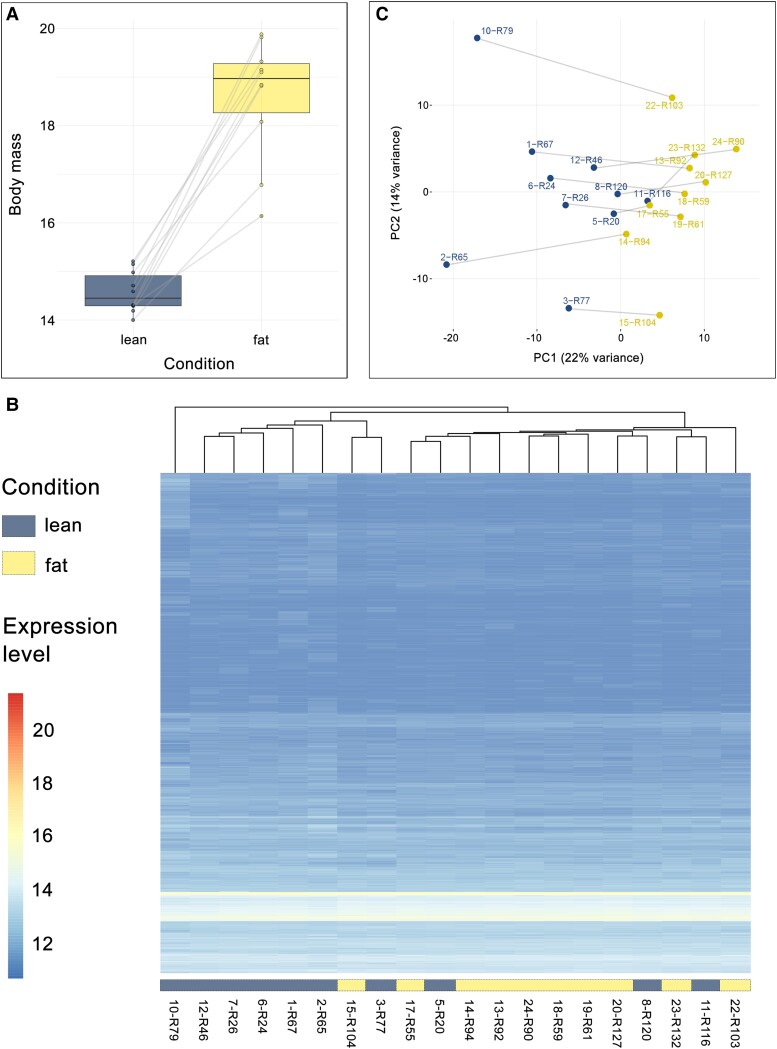
Phenotype and overall gene expression patterns in the whole blood of garden warblers. (*A*) Boxplot showing body mass measurements of individuals belonging in the two condition groups explored (lean vs. fat). Lines connect each paired sample (samples from the same individual). (*B*) Heat map showing relationships among samples based on all expression data. (*C*) Principal components (PC1 and PC2) for the gene expression data after VST. (*D*) Graphical representation of the clustering of 50 most expressed genes.

We were able to sequence the whole blood transcriptome of 10 garden warblers before and after refueling (lean vs. fat condition). All birds exhibited an increase in body condition as shown by the 28% increase in body mass (paired *t*-test *P* < 0.0001; fig. 1*A*; supplementary table S1, Supplementary Material online) thus confirming our condition classification. We expect body mass to be an accurate index of refueling condition as it exhibits a strong correlation with fat mass (Labocha and Hayes 2012). To identify patterns of differential gene expression, an average of ∼62 million reads per individual were assembled and mapped with reference to zebra finch genome, and we retrieved a total of 11,891 genes for which we were able to quantify expression levels (supplementary table S2, Supplementary Material online).

Total gene expression profiles largely reflected the two experimental conditions (fig. 1*B*); however, some within-condition variation in their expression can be observed. In fact, most individuals within each condition formed well-defined clusters, except for four samples (two individuals in both conditions) that clustered together. This within-condition variation is rather unsurprising because our opportunistic sampling of birds during migration could involve birds that originate from different natural populations. This can be further seen in the principal component analysis of all samples using all normalized expression data after variance stabilization transform (VST) that showed that the two groups differentiate in space along the PC1 axis, explaining 22% of the variation, whereas PC2 seems to differentiate the different individuals (fig. 1*C*). The topmost abundant genes expressed in the whole blood of garden warblers during migration (supplementary table S2, Supplementary Material online) have previously been implicated in the establishment of the migratory phenotype including genes involved in oxygen transport and binding, iron homeostasis, and angiogenesis.

**Fig. 2.**
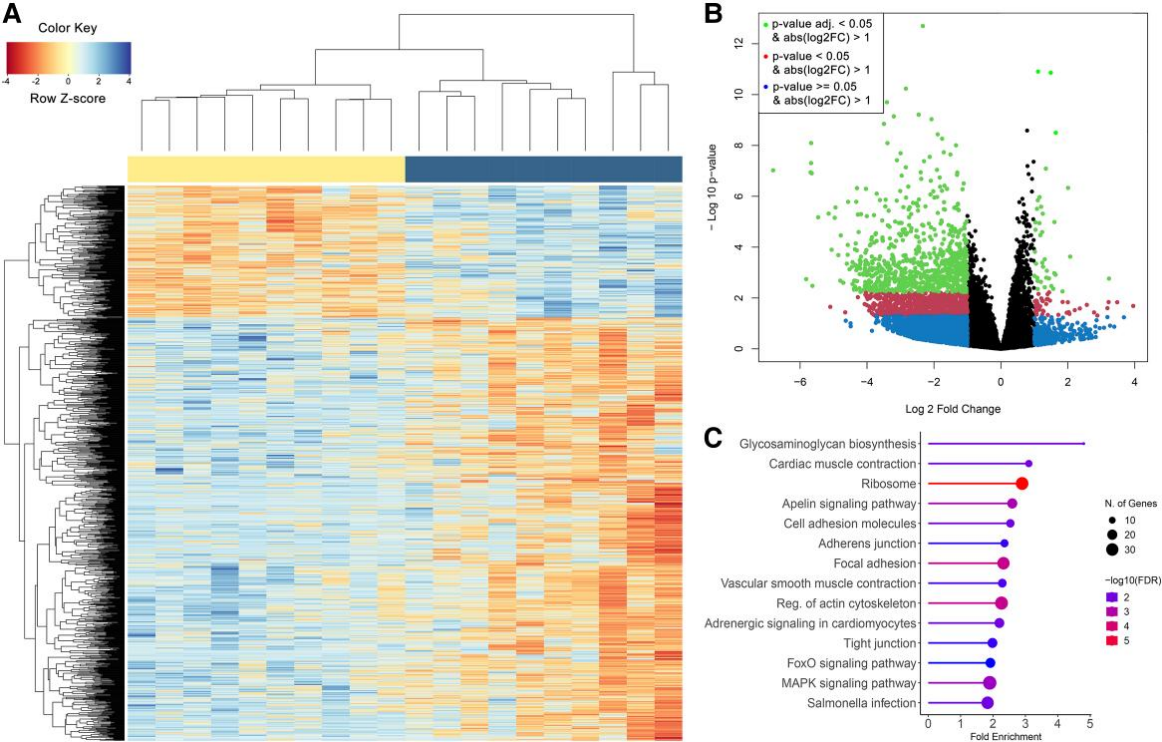
Transcriptome-wide differential gene expression analyses. (*A*) Heat map of 1,167 DEGs between the two refueling states. Scaled expression values show upregulated (positive z-score) and downregulated (negative z-score) genes. Dendrogram groups correspond to fat (left) and lean (right) refueling states. (*B*) Volcano plot of the distribution of all DEGs, depicting the log2fold change and negative log10 nominal *P*-value for all the expressed genes. Points colored green, red, and blue indicate genes with an adjusted *P* > 0.05, *P* > 0.05, and *P* < 0.05, respectively. (*C*) The top enriched KEGG pathways for DEGs. Lollipop diagrams show fold enrichment, significance (False Discovery Rate in log10), and number of genes in each pathway. The *P*-values are adjusted using Benjamini–Hochberg correction and the cutoff is 0.05. (*D*) Boxplots of DEGs (upregulated in lean state, top half; upregulated in fat state, bottom half) in the whole blood of garden warblers with the highest fold changes in expression between states.

We found 1,167 significant differentially expressed genes (DEGs) between refueling states (fig. 2*A* and *B*; supplementary table S2, Supplementary Material online), with 277 upregulated and 890 downregulated in fat birds, providing evidence for change in regulation of metabolic pathways within individuals related to refueling before continuing migration. To evaluate these transcriptional signatures, we identified several downregulated pathways (fig. 2*C*; supplementary table S3, Supplementary Material online), enriched with genes associated with glycan metabolism (glycosaminoglycan [GAG] biosynthesis; *CSGALNACT2*, *CHST15*, *CSGALNACT1*, *UST*, *DSE*, and *CHSY1*), circulatory system regulation including adrenergic signaling in cardiomyocytes, cardiac muscle and vascular muscle contraction (*ATP2A3*, *TPM4*, *TPM1*, *CACNA2D3*, *CACNA2D1*, *CACNB2*, *RYR2*, *CACNA1C*, *TPM2*, *CACNA2D2*, *ASPH*, *MAPK1*, *CALCRL*, *KCNMA1*, *PLA2G4A*, *PLCB4*, *PRKCE*, and *CALM2*), several signaling pathways involved in environmental information processing (Apelin signaling pathway, MAPK signaling pathway, FoxO signaling pathway, and Wnt signaling pathway), and genes involved in several cellular processes (focal adhesion, adherens junction, and tight junction). On the other hand, enriched pathways found to be upregulated in fat birds are involved in translation and protein processing including ribosome biogenesis (*RPL36*, *RPL38*, *MRPS17*, *RPS24*, *RPL36A*, *RPS21*, *RPL5*, *RPL27A*, *RPS13*, *MRPL28*, *MRPL2*, *RPL39*, *RPS3A*, *RPS8*, *RPL15*, *SMARCAL1*, *RPS20*, *RPS15*, *RPL24*, *RPS7*, *RPL31*, *RPLP0*, *RPS16*, *RPL21*, *RPL30*, *RPLP2*, *RPL7*, *FAU*, *RPL35*, *RPS27A*, *RPS27*, and *MRPL15*). Overall, top DEGs between conditions (fig. 2*D*) included genes upregulated in lean birds that are involved in cellular senescence (*KNDC1*), sphingolipid biosynthesis (*SPTLC3*), photoreception (*PROM1*), and transcription factors and signaling proteins (*TRPS1*, *OLFML2B*). Top upregulated genes in fat birds are involved in metabolic processes (*P4HA2* oxidoreductase, *DCAKD* involved in ATP binding, and *PDHX* in acyltransferase activity), in translation and protein synthesis (*URB2* and *SDHD*), and in organ development (*PTCD2*) including a heat shock protein (*DNAJB5*) that serves as a main regulator of heart hypertrophy, thus reflecting functional states at the two stages of migration stopover, arrival (when birds are lean) and departure (when birds are fat).

**Fig. 3.**
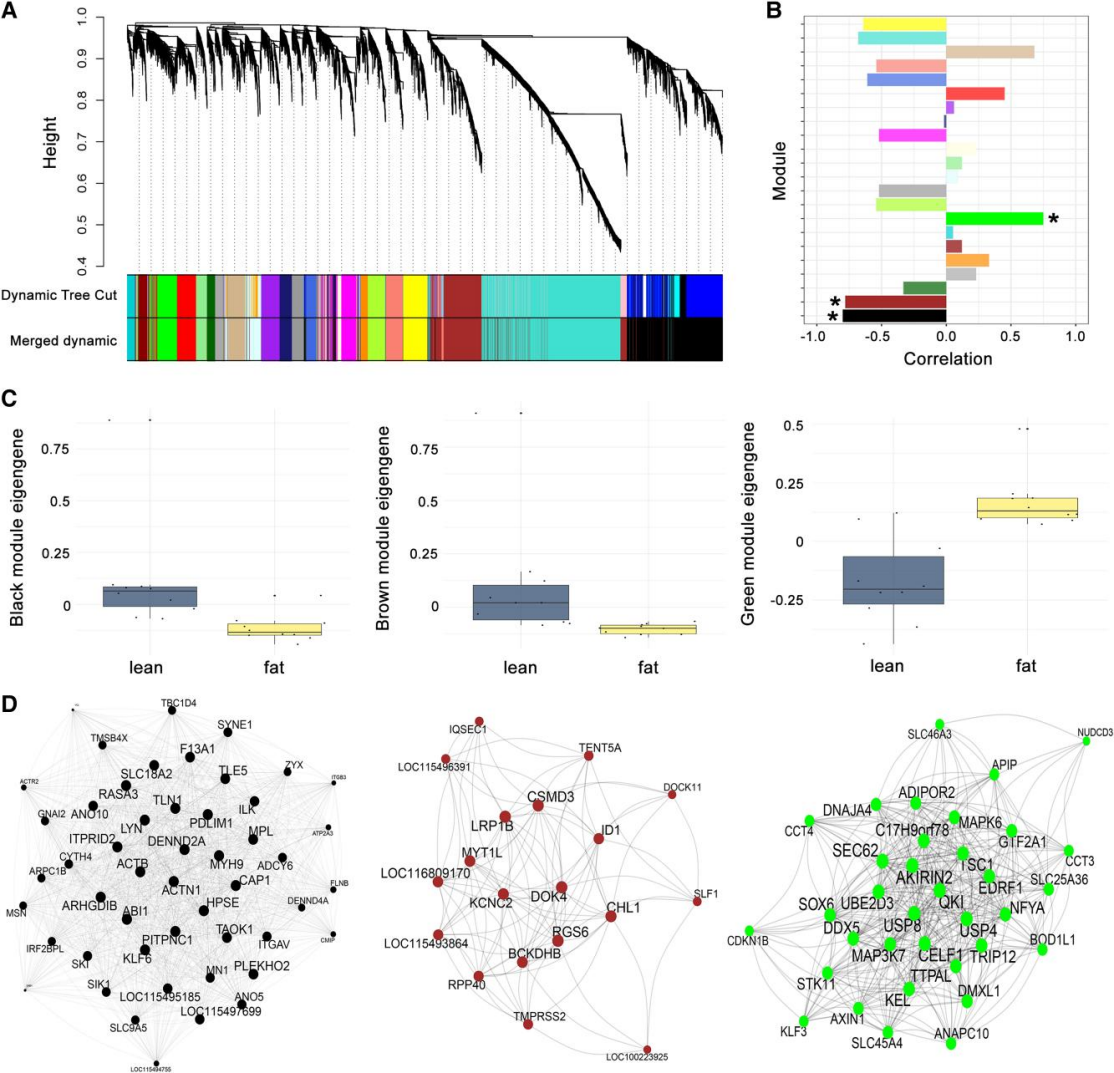
WGCNA to identify hub genes and pathways associated with different refueling states. (*A*) Dendrogram depicting clustering of genes based on 1-Topological Overlap (1-TO) distance. (*B*) Module-refueling state correlation barplot. Significant modules are indicated with asterisks. (*C*) Boxplots of relative ME expression between lean and fat states in black, brown, and green modules from left to right. (*D*) Coexpression network of hub genes (showing only genes with weighted connection >0.4 for clarity) upregulated in the lean state (black and brown modules) and upregulated in the fat state (green module).

The weighted gene coexpression network analysis (WGCNA) identified 22 coexpression modules of highly interconnected genes (fig. 3*A*). Correlation of module eigengene (ME) values with the refueling state identified three significant modules (Bonferroni correction *P* < 0.002; fig. 3*B*): The green module showed a significant positive correlation with the fat state (*r* = 0.75) with 483 genes. Black and brown modules showed significant correlation with the lean state (*r* = −0.8 and *r* = −0.78) with 2,171 and 1,367 genes, respectively (fig. 3*C*; supplementary table S4, Supplementary Material online). Genes included in the green module are involved in protein processing including ribosome biosynthesis and ubiquitin-mediated proteolysis (supplementary table S5, Supplementary Material online). Genes in the black module are mainly involved in cellular processes such as regulation of actin cytoskeleton and focal adhesion albeit coexpressed with genes involved in carbohydrate metabolism (*ADPGK*, *PKM*, *ENO1*, *ACSS2*, *HK1*, *GPI*, *PFKP*, *PGK1*, *ALDH7A1*, *ENO4*, *PGM1*, *BPGM*, *GALM*, *GAPDH*, *ALDOB*, *ALDOC*, and *PGM2*; supplementary table S5, Supplementary Material online). Finally, genes in the brown module are involved in muscle tissue development (*NOTCH1*, *HLF*, *GTF3C5*, *ALDH1A2*, *HDAC4*, *EYA2*, *MYLK2*, *EGR1*, *CHRNA1*, *GREM1*, *RYR2*, *RARB*, *MAFF*, *SAV1*, *BMP4*, *DSP*, *FGF20*, *CITED2*, *ACTN2*, *MEGF10*, *PRKAA1*, *ISL1*, *GATA6*, *BVES*, *NRG1*, and *Pax5B*; supplementary table S5, Supplementary Material online). Based on a kME > 0.8 and gene significance (GS) *P* < 0.05, we identified 38 hub genes significantly expressed in the green module, 101 hub genes in the black module, and 19 hub genes in the brown module (fig 3; supplementary table S4, Supplementary Material online). Hub genes in the green module contain 25 DEGs found to be upregulated in fat birds, whereas hub genes in the black and brown module contain 89 and 15 DEGs respectively, found to be upregulated in lean birds.

## Discussion

Changes that characterize the migratory phenotype in birds have fascinated biologists for years. However, the molecular pathways underlying the physiological changes that accompany this phenotype remain poorly understood. In our study, we chose to investigate the whole blood of migrating garden warblers because this nondestructive method allowed us to assess transcriptional changes in the same individuals during the expression of two different phenotypic states thus keeping the “background noise” to a minimum (Liedvogel et al. 2011). Despite that fact that using whole blood as a source of RNA could lead to some tissue-specific transcripts (e.g., expressed in the brain) to be missed, blood is expected to show signals of migration-related transcripts such as those involved in immune function, fueling and aerobic capacity (Fudickar et al. 2016; Jax et al. 2018), although it has been shown that it can be used to actually infer an individual's tissue-specific expression in humans, albeit with high intratissue variability (Basu et al. 2021). In our case, we detected increased hemoglobin expression levels that possibly reflect a response to increased oxygen demands of migration as shown by increased hemoglobin concentration measurements in other migratory birds (Minias et al. 2013; Butler 2016). Specifically, we did find one hemoglobin subunit, HBAA, to be differentially expressed between lean and fat birds, a result consistent with the hypothesis that during refueling, birds increase their blood parameters as an anticipation of the increased aerobic requirements of the impending migratory flight (Landys-Ciannelli et al. 2002). Furthermore, we found high expression levels of genes involved in angiogenesis, such as the *EPAS1* gene that has been related to the performance of elite endurance athletes in humans (Henderson et al. 2005; Voisin et al. 2014) and additionally found to be differentially expressed in the brain and liver of birds under different states of refueling (Frias-Soler et al. 2020, 2022) and between the different migratory seasons (spring vs. autumn; Sharma et al. 2018). Increased expression of genes involved in aerobic capacity along with angiogenesis process could be an important adaptation, especially for migrants flying at high altitudes.

Unsurprisingly, we found that genes encoding enzymes involved in different aspects of metabolic activity especially for lipid and carbohydrate metabolism were differentially expressed between refueling states. These results are consistent with previous research that found wide-scale changes in transcripts regulating metabolic pathways for lipids, carbohydrates, and protein between different migratory states (Franchini et al. 2017; Singh et al. 2018; Horton et al. 2019; Sharma et al. 2021, 2022; Frias-Soler et al. 2022). More than 30 genes associated with ribosomal protein biosynthesis were found to be upregulated in the fat refueling state, indicating a high translational capacity in the cells, thus an elevated rate of protein synthesis in preparation for migration. Staying hydrated during migration is crucial, and protein catabolism yields more endogenous water than fat; therefore, proteins represent a significant water source (Klaassen 2004). Such ribosomal genes make potentially good biomarkers that could help accurately characterize the migratory phenotype in the wild (Fudickar et al. 2016). Similarly, overexpressed genes involved in protein processing and misfolding such as heat shock proteins and ubiquitin-related genes reflect the augmentation of the metabolic activity toward departure for migration (Frias-Soler et al. 2022). In fact, most hub genes associated with fat state regulate ubiquitination processes. *UBE2D3* accepts ubiquitin from the E1 complex and catalyzes its covalent attachment to other proteins. *TRIP12* is an E3 ubiquitin-protein ligase involved in ubiquitin fusion degradation (UFD) pathway that activates *NEDD8*, a ubiquitin-like protein, while *TSC1* gene with *USP4* and *USP8* enzymes have extended functions including deubiquitination of genes (*PDPK1*, *EPRS*, *EIF4EBP1*, and *S6K1*) involved in important signaling pathways ensuring a cellular response to growth factors, specifically the mTORC1 and MAPK signaling pathways. Interestingly, *TRIP12* and two RNA-binding proteins identified among the top hub genes (*CELF1* and *QKI*) have all been associated with increased body mass index and increased body fat percentage in human genome-wide association studies (Pulit et al. 2019; Sakaue et al. 2021); thus, further investigation in future functional migration studies will certainly be of interest. Additionally, a hub gene associated with the fat refueling state, *AKIRIN2*, is a molecular adapter that acts as a bridge between a variety of multiprotein complexes and is involved in adaptive immunity by promoting B-cell activation (Tartey et al. 2015), while we also identified DE serine proteases and genes with antimicrobial activity against *Salmonella* serovars. This fact highlights that before migration, birds have to develop a strong innate immunity to cope with the continuous exposure to several pathogens (Eikenaar, Hessler, et al. 2020b). Finally, although we expected that few—if any—genes involved in circadian rhythms would be identified in our study as they are predominantly expressed in the brain, one of the top DEGs, upregulated in fat birds, was Synaptotagmin-XII (*SYT12*). *SYT12* has showed higher expression in wakefulness and sleep deprivation conditions (Jones et al. 2008) as those that would be expected from fat birds in anticipation of migration through the behavioral manifestation of migratory restlessness (or Zugunruhe; Wagner 1930).

We found that the majority of DEGs were upregulated in lean birds than in the fat state when birds have already built their reserves and are ready to migrate. Migratory strategies shape stopover ecology particularly regarding decisions of when, where, and for how long to stop. Specifically in our case, this is not just another stopover; birds arrive depleted after the long crossing of the ecological barrier (Barboutis et al. 2022); therefore, their stopover is obligatory rather than facultative. It seems that birds after long endurance flight use the stopover to first recover physiologically and then proceed to replenish their energy stores. This is supported by our DE analysis, as upregulated genes in lean birds were mostly involved in myogenesis rather than lipogenesis. But what is the scale of the recovery? Bauchinger and Biebach (2005) found that garden warblers migrating across the Sahara exhibited a decrease in heart mass and flight muscle mass that was restored after a 9-day refueling. Based on our results, five genes belonging to the calcium channel gene family seem to be involved in the regulation of Ca^2+^ channels, three genes in the tropomyosin family bind to actin filaments in muscle and nonmuscle cells, and *ASPH* gene is involved in calcium homeostasis along with ryanodine (*RYR2*) that further activates *KCNMA1* in smooth muscles, initiating contraction (Catterall 2011). From these genes, *CACNA1C* function includes normal heart development and normal regulation of heart rhythm and is required for normal contraction of smooth muscle cells in blood vessels and in the intestine. It is therefore highly plausible that such groups of genes could be involved in this extraordinary phenotypic plasticity in organ mass. Hub genes highly associated with lean birds include several candidate genes (*MYT1L* transcription factor, *CSMD3*, *KCNC2*, *CHL1*, and *BCKDHB*) for lean body mass in humans (Locke et al. 2015; Tachmazidou et al. 2017; Pulit et al. 2019).

Our results provide further insight on adaptations related to stopover refueling after barrier crossing by identifying upregulated genes when birds arrive to the stopover. We draw attention to the emerging role of two novel pathways that seem to be involved in recovery after endurance flight, namely, the apelin signaling pathway and GAG biosynthesis. GAGs play a crucial role in cell signaling processes including regulation of wound repair (Raman et al. 2005; Melrose 2016). Changes in structure and bioactivity of GAGs, especially heparan sulfate (HS) and chondroitin sulfate (CS), can affect signaling pathways of myogenesis, although it has been shown that GAG mimetic treatment accelerated muscle repair after ischemia in rats (Chevalier et al. 2015), thus highlighting the role GAGs can play in tissue engineering (Sodhi and Panitch 2020). Such efficient biochemical and physiological mechanisms could therefore provide a defense against muscle damage that occurs during migration (Guglielmo et al. 2001). Overall, genes included in the abovementioned pathways and the related metabolites are candidates for further functional studies. Furthermore, due to their high polarity, GAGs may play a crucial role in water homeostasis, while defective GAG synthesis can lead to abnormal water balance (Olde Engberink et al. 2019) whereas apelin is implicated in several physiological processes with many studies supporting its role in the maintenance of body fluid balance by regulating water reabsorption, as well as influencing urine volume interacting with the antidiuretic hormone, AVP (Hu et al. 2021). This pathway may represent a significant adaptive mechanism, as in order to conserve water during long-distance flight, birds do get some water supply from protein catabolism but due to resulting negative nitrogen balance, nitrogen is converted to uric acid that requires little urinary water (Ramenofsky and Wingfield 2007).

Here, we show that a whole blood transcriptomic approach could be used to successfully study expression in the same individuals at different states, as we were able to identify genes that were previously found to be expressed in the brain and liver of studied songbirds. Furthermore, we provide additional genes involved in a wide range of functions and identify novel pathways that could be used to build hypotheses to be tested in future approaches. The more functional studies on bird migration, the more obvious it becomes that the migratory phenotype is not the result of a single adaptation or some few “migratory” genes. A suite of different and often complex adjustments in morphology, metabolism, biomechanics, and behavior take place, so it is expected that the molecular basis of such changes is most probably very complex too. Still, only a few studies exist that address differential expression of genes at different stages of migration. More studies and a synthetic approach are needed to further identify mechanisms that allow avian migrants express their remarkable phenotype.

## Materials and Methods

### Sample Collection and Experimental Setup

Ten migrating garden warblers were captured during spring migration of 2022 (April 20 to May 7) using 16 × 16 mm mesh, nylon, mist nets in Antikythira Island, an important migration stopover site (Barboutis et al. 2022), located in the Eastern Mediterranean (35° 51′ N, 23° 18′ E), within the standardized monitoring program of migratory birds run by the Hellenic Ornithological Society and the Hellenic Bird Ringing Centre. Birds were ringed, measured, and weighted to the nearest 0.1 g according to Svensson (1992) and then housed in individual cages in natural conditions for 5 days, where they were provided with food (mealworms, *Tenebrio molitor*) and water ad libitum. We selected lean birds for collection to represent individuals that would use the island as a stopover to refuel and not resume their migration during the upcoming night, based on their body mass measurements. Mean arrival body mass of garden warblers caught on Antikythira Island during spring is estimated at 16 g (Barboutis et al. 2011). We therefore kept lean birds that weighted between 14.0 and 15.2 g (fig. 1*A*) in individual cages measuring 50 × 30 × 30 cm, built from nonmagnetic material (PVC and wood) while lined in all sides with white semitransparent cotton fabric to provide natural light conditions. Every night, the cages were enclosed in a tent, and the mean night temperature was 15.7 ± 1.3 °C for the whole duration of the experiment. Body mass was recorded early in the morning daily before the birds received food. Blood samples (50 *µ*l) for transcriptomic analysis were collected from each bird twice, on the first and fifth day (shortly before migratory birds were released) during morning hours (8:00–10:00 h), and were obtained by puncturing a brachial wing vein with a 25-gauge needle. Blood was immediately stored in DNA/RNA Shield buffer (Zymo Research, Orange, CA, USA) that was initially kept at room temperature until shipment (within less than a month from collection) and then preserved at −20 °C until RNA extraction, following Komini and Bounas (2022). Capturing and sampling of birds were performed under the license 113670/3168 issued by the Hellenic Ministry of Environment and Energy. This study was carried out in accordance with the European Convention for the Protection of Vertebrate Animals Used for Experimental and Other Scientific Purposes of the Council of Europe (http://conventions.coe.int/Treaty/EN/Treaties/Html/123.htm).

### RNA Purification and Sequencing

The Quick RNA Whole-Blood Kit (Zymo Research, Orange, CA, USA) was used for RNA purification following the manufacturer's guidelines. RNA concentration, purity, and integrity were evaluated using standard spectrophotometric measurements and 0.9% agarose gel electrophoresis, whereas overall quality was measured through an Agilent 2100 Bioanalyzer RNA assay (Agilent Technologies, Santa Clara, CA, USA). High-quality RNA samples (RIN = 8; 260/280 ratio = 1.8) were sequenced at IGA Technology Services, Udine, Italy, on paired-end 150 bp mode on NovaSeq 6000 (Illumina, San Diego, CA, USA). Universal Plus mRNA-Seq kit (Tecan Genomics, Redwood City, CA, USA) was used for library preparation following the manufacturer's instructions (library type: fr-secondstrand).

### Quality Filtering and Mapping

We obtained 1.52 billion raw reads (33–121 million reads per sample; average 64 million reads per sample). Raw reads were demultiplexed, and adapters were masked with Illumina BCL Convert v3.9.31 (https://support.illumina.com/sequencing/sequencing_software/bcl-convert/documentation.html). Reads were trimmed with ERNE 2 (Del Fabbro et al. 2013) and then subjected to quality control using FastQC (Andrews 2010) with a passing score threshold *Q* > 30. Further exploration of quality control was performed using RSeQC (Wang et al. 2012). We used STAR (Dobin et al. 2013) with default parameters to map reads to the latest zebra finch genome (*Taeniopygia guttata*, release 106, accession number: GCF_003957565.2), and quantification of gene expression was performed with StringTie (Pertea et al. 2015) by calculating the fragments per kilobase per million (FPKM) of each gene.

### Statistics

Differential gene expression analysis between test groups was performed using HTSeq (Anders et al. 2015) to preprocess RNA-seq data for differential expression analysis by counting the overlap of reads with genes. The package DESeq2 (Love et al. 2014) was then used to perform comparisons between expression levels of genes and transcripts by fitting a generalized linear model (GLM) for each gene. This tool uses shrinkage estimation for dispersions and fold changes to improve stability and interpretability of estimates. Normalization was performed using the median-of-ratio method (Anders and Huber 2010), and statistical significance was determined using a Wald test (Love et al. 2014). A *P*-adjusted value of 0.05 was used as a significance cutoff threshold. Based on expression patterns, DEGs were categorized as upregulated or downregulated in each refueling state. We determined which Gene Ontology (GO) terms were overrepresented among DE genes using ShinyGO (Ge et al. 2020), based on the GO–biological process (no-redundant), GO–molecular function (no-redundant), and Kyoto Encyclopedia of Genes and Genomes (KEGG) pathway databases (Kanehisa et al. 2021). We required at least five significant genes within a GO term to be reported to avoid biases caused by GO terms with small numbers of genes and used a 0.05 *P*-value cutoff (Fudickar et al. 2016). We used the identified 11,891 genes from this RNA-seq experiment as a background gene set for the analysis and selected the zebra finch as model species. All *P*-values were corrected for multiple testing using the Benjamini–Hochberg method.

We further used normalized FPKM values to perform WGCNA and get an unbiased assessment of coexpression of genes significantly associated with refueling state (lean vs. fat), using the WGCNA package in R (Langfelder and Horvath 2008). Signed networks were constructed with the following parameters: deepSplit = 2, minimum module size = 30 and minimum cutoff height of 0.25. A soft-power threshold of eight was selected under the scale-free topology criterion (Zhang and Horvath 2005). The ME is considered as the representative of a module's gene expression profile and is defined as the first principal component of gene expression values in the given module. We calculated ME values based on all genes in each module and performed Spearman correlations with the two-level factor state (lean and fat). Modules exhibiting absolute correlation above 0.7 and a *P* < 0.002 (adjusted for multiple comparisons) were identified as target modules. The biological function of genes belonging in each target module was explored through enrichment analysis, and hub genes for each of the three target modules were chosen based on a module membership (kME) value > 0.8 and statistically significant GS. Gene interaction networks were visualized with “ggraph” R package (Pedersen 2021). The R programming language platform (version 4.2) was employed for statistical analysis and plotting (R CoreTeam 2022).

## Supplementary Material

evad061_Supplementary_Data

## Data Availability

The sequence data are available in the NCBI Gene Expression Omnibus repository and are accessible through GEO accession number GSE225337.
